# Theta phase precession of grid and place cell firing in open environments

**DOI:** 10.1098/rstb.2012.0532

**Published:** 2014-02-05

**Authors:** A. Jeewajee, C. Barry, V. Douchamps, D. Manson, C. Lever, N. Burgess

**Affiliations:** 1 Department of Cell and Developmental Biology, UCL, London WC1E 6BT, UK; 2 Centre for Mathematics and Physics in the Life Sciences and Experimental Biology, UCL, London WC1E 6BT, UK; 3 Institute of Cognitive Neuroscience, UCL, London WC1N 3AR, UK; 4 Institute of Neurology, UCL, London WC1N 3BG, UK; 5 Department of Psychology, University of Durham, Durham DH1 3LE, UK

**Keywords:** temporal coding, theta rhythm, hippocampus, entorhinal cortex, space, oscillation

## Abstract

Place and grid cells in the rodent hippocampal formation tend to fire spikes at successively earlier phases relative to the local field potential theta rhythm as the animal runs through the cell's firing field on a linear track. However, this ‘phase precession’ effect is less well characterized during foraging in two-dimensional open field environments. Here, we mapped runs through the firing fields onto a unit circle to pool data from multiple runs. We asked which of seven behavioural and physiological variables show the best circular–linear correlation with the theta phase of spikes from place cells in hippocampal area CA1 and from grid cells from superficial layers of medial entorhinal cortex. The best correlate was the distance to the firing field peak projected onto the animal's current running direction. This was significantly stronger than other correlates, such as instantaneous firing rate and time-in-field, but similar in strength to correlates with other measures of distance travelled through the firing field. Phase precession was stronger in place cells than grid cells overall, and robust phase precession was seen in traversals through firing field peripheries (although somewhat less than in traversals through the centre), consistent with phase coding of displacement along the current direction. This type of phase coding, of place field distance ahead of or behind the animal, may be useful for allowing calculation of goal directions during navigation.

## 1. Introduction

Place cells [1] and grid cells [2] recorded in the hippocampus and medial entorhinal cortex of freely moving rodents have spatially modulated firing patterns. Place cells typically fire whenever the animal enters a single firing field, whereas grid cells fire whenever the animal enters any one of an array of firing fields distributed across the environment at the vertices of a regular triangular grid. (In larger environments, place cells also have multiple fields, but the fields lack periodicity [3], see also [4].) In addition to their spatially modulated firing rates, place cells [5] and grid cells (at least those in layer II [6]) also show a form of temporal or phase coding relative to the ongoing theta rhythm of the local field potential (LFP). Whenever a rat or mouse is involved in translational motion, the LFP shows a large amplitude oscillation which is typically around 7–9 Hz in an adult animal [7–9]. When the animal runs through the firing field of a place or grid cell, the cell typically fires short bursts of one or more action potentials (‘spikes’) whose phase relative to the LFP theta shifts systematically from later to earlier phases, a phenomenon known as theta phase precession [5,10]. The spikes are fired at the peaks of a theta-band membrane potential oscillation which shows phase precession relative to the LFP, in both place [11] and grid cells [12,13].

Much of the interest in theta phase precession comes from the observations that firing phase appears to code for distance travelled through the field rather than, for example, time spent in the field [5], and that firing phase contributes information about the animal's location beyond that encoded by firing rate alone [14]. This suggests that the temporal dynamics of firing varies between slow runs and fast runs so that phase of firing codes for distance travelled (see also [15]). However, the exact nature of the variable or variables that might be encoded by theta phase precession and the physiological mechanisms causing it have been the subject of intense debate. In addition, most research has focused on rats running along linear tracks (but see also [10,16,17]). In these simplified linear environments, in which running patterns often become stereotyped, theta rhythmicity tends to be clearer than that during free exploration of open (two-dimensional) environments, but independent variation of directions, speeds and firing rates is much reduced.

**Figure 1.**
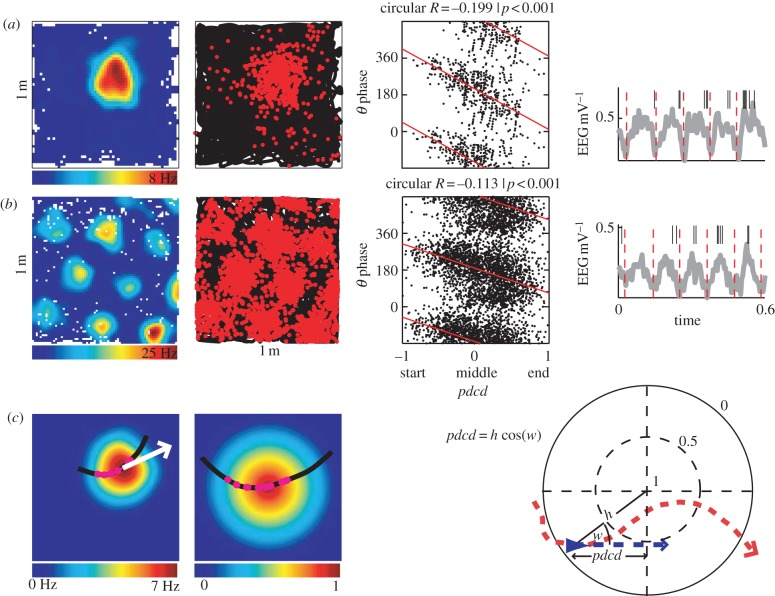
Mapping runs through fields onto the unit circle. (*a*) Example of phase precession in single place cell showing the firing rate map (left), the spikes as dots on the animal's path (black line; left middle), raster plots of spike phase versus *pdcd* (circular–linear regression line shown; right middle) and an example of the timing of spikes (vertical ticks) versus LFP (grey line, right). (*b*) Example of phase precession in a single grid cell (organized as in *a*). (*c*) Example of the transformation of a run through a firing field onto a unit circle which represents the firing field with the peak firing rate at the centre. Locations on the run are transformed radially so that their proportional distance between the firing peak and the perimeter is preserved, and then rotated about the centre of the circle so that the mean direction of the run (white arrow) is left–right for all runs; see text for details. *Pdcd* is the distance of the animal to the field peak projected on the current running direction, which varies from −1 to 1 as the animal enters the field on the left and exits on the right. (Online version in colour.)

Suggested mechanisms for phase precession range from phase advancing owing to increasing depolarization [18–20], or owing to the after-hyperpolarization/depolarization dynamics following the firing of a previous spike [21], to temporal integration of speed-related changes in burst frequency to compute distance travelled [15,22], or temporal integration of velocity-related changes in burst frequency to compute displacement along the current direction [23–26]. This last measure equates to phase coding of the distance of the field centre ahead of or behind the rat, which has been shown to correlate with firing phase in two dimensions [16], and is closely related to the ‘directional rate zone’ shown to correlate with firing phase [17] (figure 1).

Here, we consider the simple question of which behavioural or physiological variables show the best circular–linear correlation with the theta phase of spikes from place cells and grid cells as rats forage in open two-dimensional environments. We then attempt to visualize the spatial distribution of firing phase across the firing field. The results should give an insight into the types of information that a downstream receiver could most easily decode with a simple linear mapping from the theta phases of the spikes of one of these cells. Consideration of the very many possibilities for more sophisticated decoders—for example, decoders that can be adjusted run-by-run [27,28] or that receive a filtered subset of the spikes from a cell (e.g. those forming a ‘burst’ or ‘complex spike’ [29]); or the leading spike per theta cycle [28] or that receive spikes from multiple cells—are beyond the scope of this paper (for further discussion, see [30]).

We use a method for mapping spatial firing fields onto a unit circle aligned to the location of the peak firing rate, so that data from individual runs can be accumulated according to their position within the firing field relative to the direction of the run through the field (figure 1). This provides a common framework for analysing the spatial characteristics of all runs through the firing field of a place cell or through the multiple firing fields of a grid cell. We first examine which variables best correlate with firing phase, including cumulative variables experienced within a normalized firing field relating to distance travelled, time, firing rate and the location of the field centre projected onto the mean direction of the run or the current direction of the animal (figure 1). We then examine whether our initial conclusions are supported by categorical analyses of the data, comparing fast or slow runs, runs containing high or low firing rates and runs through the middle or edge of the firing field. Finally, we show the two-dimensional spatial distribution of firing phase within the normalized firing field.

## 2. Material and methods

Entorhinal cortex data from six male Long Evans rats from [31], two male Lister Hooded rats [32] and 10 additional animals implanted according to the protocol in [32], are reported in this study. CA1 data were recorded from six male Lister Hooded rats [33] and two animals from [34]. Complete details of surgery, animal housing and training can be found therein. Briefly, all animals received tetrode implants to isolate individual cells and LFP was recorded locally. Rats foraged for randomly scattered food reward in a familiar, 1 m square environment, in the presence of a directional cue in either 10, 15 or 20 min trials; a 0.6 m square environment was used in some of the place cell recordings. Cells were recorded from the superficial layers of the entorhinal cortex or the hippocampal CA1 pyramidal layer.

The firing field of a place cell or the several firing fields of a grid cell were isolated by *k*-means clustering the spikes in space. Spikes and dwell time were binned at the resolution of the camera, smoothed with a Gaussian filter with standard deviation of 4 cm and divided to give the firing rate map. Regions of firing below 10% of the peak firing rate in the field were set to zero; the remaining region defined the firing field perimeter. Partial grid cell firing fields occluded by the edge of the environment were excluded, i.e. fields at the edge of the environment were required to have an area greater than three quarters of the average field area. Entries and exits from this region defined runs through the field. Qualifying runs had to maintain their speed above 2.5 cm s^−1^ throughout. All cells had to contribute more than 25 spikes after exclusion criteria had been applied, to qualify for further analysis. Then the animal's trajectory from each run through the firing field was rotated so that the average movement direction was left-to-right, and the points along it placed within the unit circle so as to maintain their angle from the field peak relative to the mean run direction, and their proportional distance between the peak and the edge of the field. To be precise, the animal's position in the field (*x*, *y*) was transformed into normalized polar coordinates in the unit circle (*r*, *θ*) via three intermediary vectors ***v***, ***p*** and ***q***, where ***v*** is the mean velocity vector of the run, ***p*** is the vector from (*x*, *y*) to the peak of the field and ***q*** is the vector from the edge of the field to the peak of the field that passes through (*x*, *y*). Then, *r* = |***p***|/|***q***| and *θ* is the angle between ***v*** and ***p***.

Spatial maps of firing phase (500 pixels in diameter) show spike location after the above transformation and are coloured to represent firing phase by the hue of a circular colour map. The average firing phase of each pixel is the circular mean over many spikes, each of which contributes a unit vector in the direction given by its phase. However, the two orthogonal components of these vectors are then independently spatially smoothed (Gaussian kernel with s.d. 10 pixels) and the direction of the resulting vector is displayed in the two-dimensional colour plots of phase. The magnitude of the vector is used to determine the circular variance in firing phase (1-mean vector length) which is shown in colour plots of phase variance.

Phase was obtained using the Hilbert transform of the smoothed LFP; see [34] for details. Modulation of the concurrent LFP and the spike train at theta frequencies were scored by dividing spectral power density in a 2 Hz interval centred on the peak frequency in the theta band (6–12 Hz) by that across the entire spectrum (0–125 Hz). To be ‘theta modulated’ required this index to be greater than 10 for LFP and five for the spike train autocorrelogram; see [33] for detailed methods. As electroencephalograph (EEG) data from a standard reference location were not available for every cell, we attempted to normalize absolute values of firing phase across cells as follows. Each cell's spikes were binned into 1° bins according to their firing phase, the distribution was smoothed with a circular Gaussian filter with a standard deviation of 45°, and the phase at which the minimum number of spikes were fired was defined as phase zero. In addition, for plots showing phase precession combined across cells (figures 4–6), phase values across cells were normalized by setting the mean phase at the field centre to be 180° (i.e. the value of the circular linear regression of phase versus position at *pdcd* = 0 was set to 180°). Density plots of firing phase versus *pdcd* (figures 5 and 6) show the density of spikes at a given value of phase and *pdcd* per second of occupancy of that value of *pdcd*.

The ‘gridness’ of a grid cell's spatial firing pattern was determined from the spatial autocorrelogram using procedures described in [32]: ‘grid cells’ exceeded a gridness threshold of 0. The Kullback–Liebler (KL) divergence (versus a uniform distribution) was used to assess the extent to which each cell's firing was modulated by head direction using the cell's directional firing rate distribution adjusted for inhomogeneous sampling; see [35]. A cell was considered to be ‘directionally modulated’ if the KL divergence was more than 0.1, a value that provided good accordance with the experimenter's judgement. Conjunctive cells, i.e. cells with directionally modulated grid-like firing [36], were not included in the analyses. Thus, only grid cells without directionally modulated firing were included, matching the absence of directional modulation of place cell firing during foraging in open environments [35,37,38].

Runs through the centre or the periphery of the field were defined by whether more than 50% of the transformed and rotated run fell into a central region defined by two horizontal chords of the unit circle (transformed field). The distance from the centre of these two chords was chosen, for each cell, so that half of the spikes from that cell fell in the central category (and half into the peripheral category). Finally, instantaneous firing rate was calculated by smoothing the number of spikes fired per sampled position by a Gaussian filter with a standard deviation of 0.5 s.

**Figure 2.**
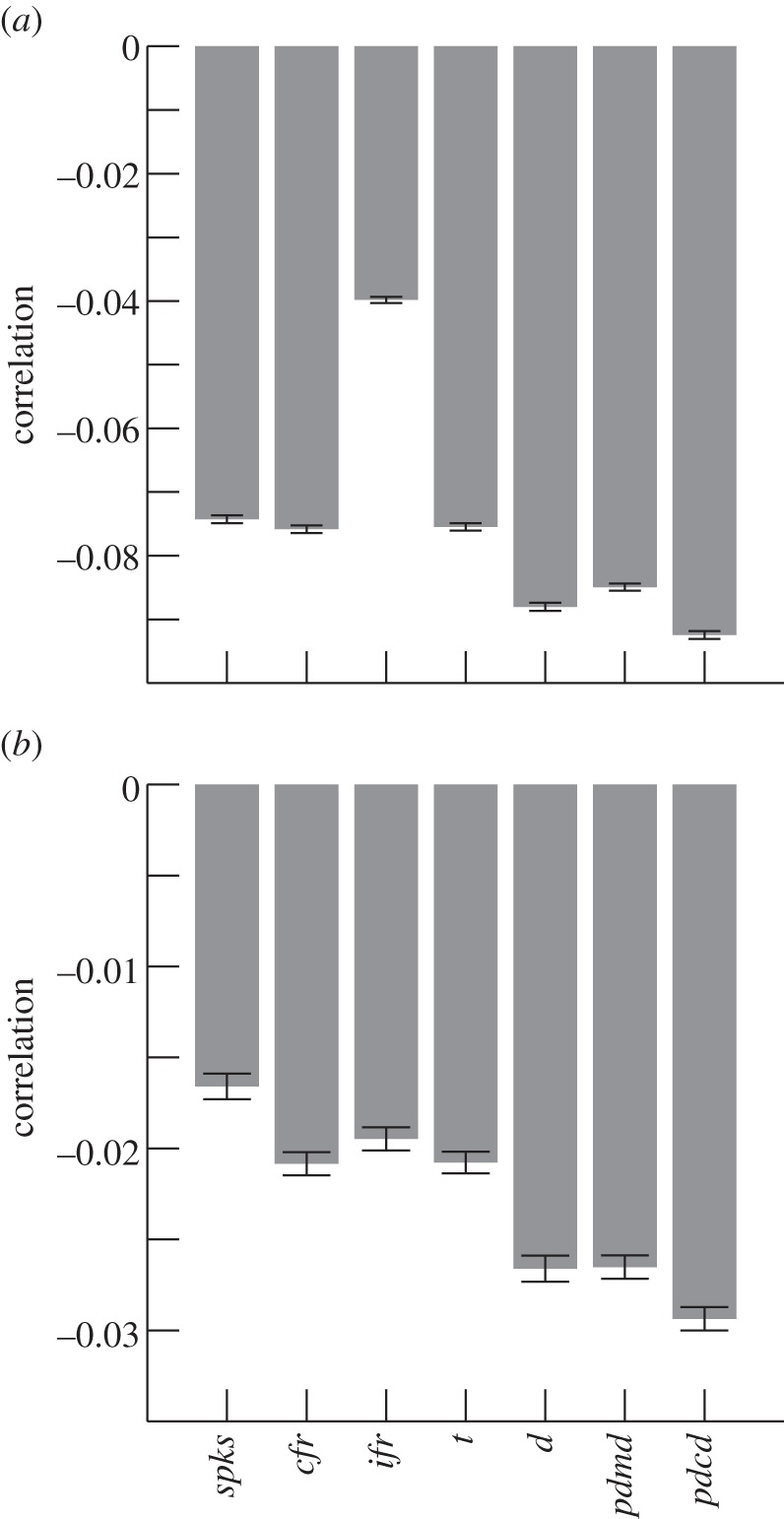
Circular–linear correlations of spike phases versus behavioural and physiological variables in spikes from populations of CA1 place cells (*a*) and grid cells from superficial layers of medial entorhinal cortex (*b*). Bar charts show the mean and standard error of the correlation coefficient.

**Figure 3.**
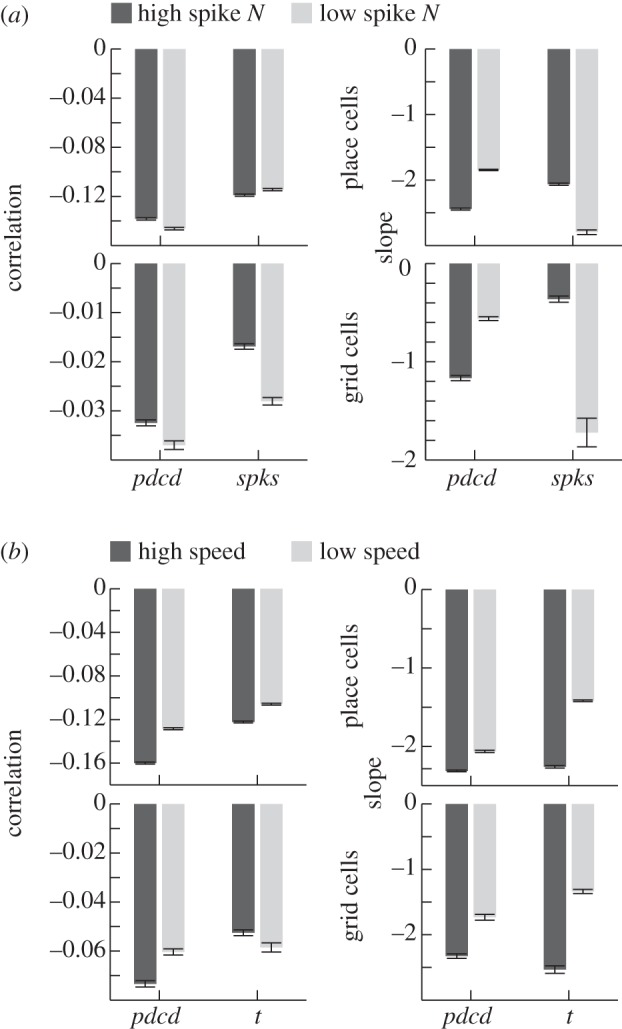
Effects of firing and running speed on phase precession, in cells that show significant phase precession versus *pdcd* (place cells above and grid cells below). Showing the circular–linear correlation coefficients and regression slopes of firing phase versus *pdcd* and *spks* for low and high firing runs (*a*), and firing phase versus *pdcd* and time for slow and fast runs (*b*). Slopes are given ‘per unit *x*’ where *x* is *pdcd* or time (*t*), and ‘per 10 spikes’ for phase versus *spks*, for clarity.

Details of the circular statistics used in the analyses, including circular–linear regression and correlation can be found in sections 6.3-4 of [39]. Similar results, with larger absolute correlation values, are obtained using the alternative method in [40]. All analyses were conducted using Matlab (Matlab Release 2008b, MathWorks, Natick, MA, USA). Cells were considered to show significant phase precession if the phase change across the field indicated by the regression line exceeded 90° and their correlation coefficient was more negative than the 97.5th percentile of the shuffled distribution of phase versus the independent variable (e.g. distance travelled). The significance of differences in the correlation coefficient or slope of a cell's firing phase versus one or other variable (as in figure 2) or in one or other group of runs (as in figures 3 and 6) were assessed by comparing the mean over cells of the within-cell difference in values to a distribution obtained by randomly changing the sign of the differences (i.e. randomizing the membership of the category of run or variable).

## 3. Results

We identified all of the continuous runs through a firing field, then we mapped the locations of the run onto a unit circle in which the centre of the circle represents the peak firing location and the mean direction of the run is left-to-right, maintaining their radial position as a proportion of the distance from the peak to the edge of the field. We examined, cell-by-cell, the circular–linear correlation between the phase of spikes fired during these normalized runs and several of the variables that might be expected to correlate with firing phase, as listed below; see §2 Material and methods for details. The variables we considered, calculated in terms of the normalized runs on the unit circle, were:

(1) number of spikes fired on the run so far (*spks*);
(2) cumulative expected amount of firing (integral of the firing field height along the path; *cfr*);
(3) instantaneous firing rate (*ifr*);
(4) time spent on the run so far (*t*);
(5) distance travelled on the run so far (*d*);
(6) distance to the field peak projected onto the current direction (*pdcd*) and
(7) distance to the field peak projected onto the mean direction of the run (*pdmd*).

It can be seen that variables 1–3 are quantities that might potentially have some physiological analogue within the cell at the time the spike was fired. Variables 4 and 5 reflect temporal and spatial elements of the animal's recent movement through the firing field prior to the firing of the spike; note that the distance travelled, *d*, is relative to the size of the firing field, as it is calculated from the normalized runs. Variables 6 and 7 combine both physiologically and behaviourally relevant quantities, see figure 1*c* for a diagram illustrating variable 7.

### (a) The relationship of firing phase to behavioural and physiological variables

Figure 2 shows the mean and standard deviation of the circular–linear correlation of firing phase with the seven behavioural and physiological variables, for the whole populations of CA1 place cells and superficial layer medial entorhinal cortex grid cells (not including conjunctive cells). The strongest overall correlation of firing phase was with the projected distance onto the current direction (*pdcd*) for both place cells and grid cells.

The proportion of cells with significant negative correlation coefficients for each of these variables, across cells, is given in table 1. A large proportion of place cells showed a significant correlation between firing phase and *spks*, but the strength of this relationship was much weaker (figure 2) and its slope much more variable across runs (figure 3*b*) than the correlation with *pdcd*. A one-way ANOVA shows that there are significant differences in the strengths of the correlations with different measures (*F*_89,5_ *=* 3.06, *p* < 0.006, for grid cells and *F*_161,5_ = 18.52, 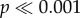, for place cells). Further permutation tests show significant differences in the strength of the correlation of phase with *pdcd* compared with the other variables. Correlations with *pdcd* were significantly stronger than that with all other variables except for *pdmd* and *d* (all *p* < 0.005 for place cells and *p* < 0.05 for grid cells).

**Table 1.** Proportions of place and grid cells showing theta phase precession.

regressor	place cells (*N* = 166) significantly phase precessing (%)	grid cells (*N* = 90) significantly phase precessing (%)
*spks*	70	36
*cfr*	58	40
*ifr*	37	30
*t*	60	39
*d*	49	38
*pdmd*	60	47
*pdcd*	61	48

Place cells, as a population, show stronger phase precession than grid cells, consistent with the observation that a significant proportion of grid cells from superficial layer III do not show phase precession [6], whereas the vast majority of CA1 place cells do [5,10,17]. Supporting this interpretation, we performed similar analyses of datasets of grid cells identified as from layer II or layer III (data from the Moser laboratory [36]), which showed that the proportion of grid cells in layer II with significant phase precession in two dimensions is higher than in layer III, and similar to the proportion of CA1 place cells. Nonetheless, the grid cells which do show significant phase precession also did not show as strong a relationship between firing phase and *pdcd* as the place cells that show significant phase precession; see for example figure 3.

### (b) Effects of run-type on firing phase in phase precessing cells

Having determined that the distance to peak projected onto the current direction (*pdcd*) provides the best overall correlate of firing phase in the populations of place and grid cells, among the behavioural and physiological variables tested (figure 2), we can use this variable to illustrate various features of the phase precession effect. First, in those cells whose firing shows theta phase precession versus *pdcd*, we examined why the other behavioural and physiological measures showed a weaker overall correlation with firing phase than *pdcd*. To do this, we divided the spikes from each cell into halves corresponding to dichotomous categorizations of different types of runs.

To examine the correlation of firing phase with the number of spikes fired on the run so far (*spks*), we divided runs into runs in which high or low overall numbers of spikes were fired. This shows that, in addition to the lower correlation values between firing phase and *spks* compared with *pdcd* (figure 3*a*, left), there is a much greater difference in the slope of the relationship of firing phase to *spks* across high and low firing runs than with *pdcd*, which is most pronounced for grid cells, see figure 3*a* (right). If firing phase shifted with each spike fired on the run so far, then we would have expected the relationship between phase and *spks* to have a constant slope across low and high firing runs. The observed variation in the slope argues against a simple relationship between phase and *spks*, especially for grid cells. And a smaller contribution of number of spikes fired to changes in firing phase, compared with the larger contribution of distance travelled (specifically *pdcd*), is consistent with previous findings for place cells on linear tracks [41].

To examine the correlation of firing phase with the time spent in the field on the run so far (*t*), we divided the runs into slow and fast runs. This shows that the relatively weak correlation between firing phase and time spent in the field on the current run (figure 3*b*, left) results in part from the different slopes of the relationship between firing phase and time seen across slow and fast runs (figure 3*b*, right). As with the potential relationship between phase and *spks*, the variation in the slope of the relationship between phase and time across slow and fast runs argues against a simple relationship between phase and time spent in the firing field.

### (c) Spatial distribution of firing phase in phase precessing cells

**Figure 4.**
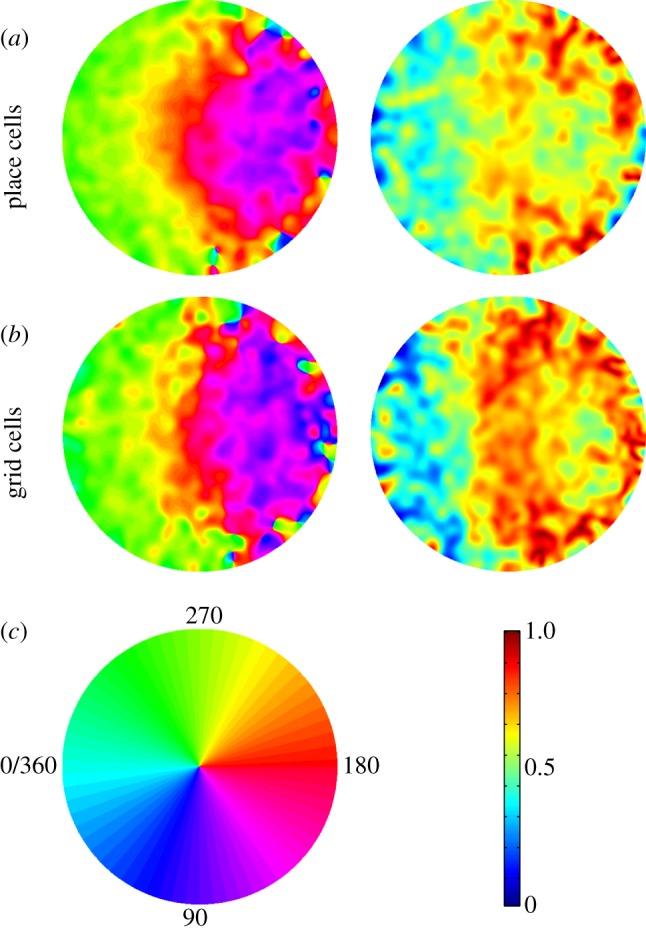
Two-dimensional distribution of firing phase (left) and circular phase variance (right) in CA1 place cells (*a*) and superficial layer medial entorhinal cortex grid cells (*b*) that show significant phase precession versus *pdcd*. (*c*) Key for phase (left) and circular variance (right). (Online version in colour.)

We now attempt to visualize the patterns of firing phase in those cells showing significant phase precession. We can investigate the two-dimensional spatial distribution of firing phase of these cells, given our mapping of all runs through a firing field onto a unit circle (figure 4). These plots show how phase and phase variance are distributed over the firing field for grid cells and place cells. In place cells and grid cells, phase reduces through the field. There is also a marked increase in the variance of firing phase from the first half to the second half of the field. Phase appears to change most rapidly at the halfway point (where the projected distance to the peak changes sign on the left-to-right runs) and the phase variance increases at this point, a pattern that is most apparent in grid cells.

**Figure 5.**
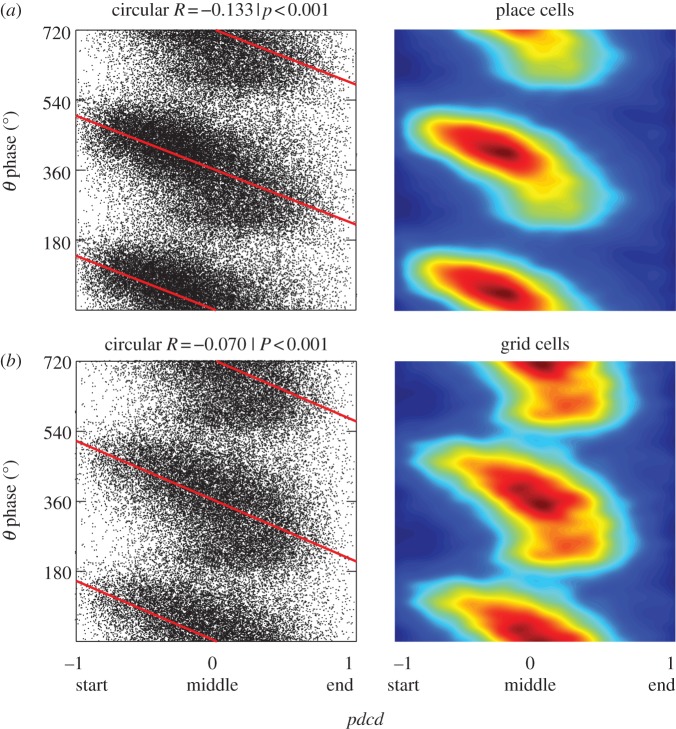
Pooled data: firing phase versus distance to field peak projected onto mean direction (*pdcd*) for place cells (*a*) and grid cells (*b*) that show significant phase precession. Raster plots and line of best fit (circular–linear regression; left), phase versus *pdcd* in occupancy-normalized density plots (right). (Online version in colour.)

The decreasing phase from left-to-right (i.e. in the mean direction of runs through the firing fields) in figure 4*a*,*b* is consistent with the relatively high correlation between phase and distance to peak projected onto the mean direction of the run (*pdmd*) in our analyses of all cells, and its similarity to the correlation with distance to peak projected onto the current running direction (*pdcd*); see figure 2. The one-dimensional equivalents of these plots (phase versus *pdcd*) are shown in figure 5. For place cells, the average change in phase across the field is 296° (with 95% confidence interval 274–324°). For grid cells, the average change in phase across the field is 310° (with 95% confidence interval 273–325°).

Note that a small proportion of phase precessing cells show a significant positive rather than negative correlation between firing phase and *pdcd* (i.e. a positive correlation greater than the 97.5 percentile in the shuffled distribution; 4% of place cells, 20% of grid cells). As noted in [6], these tend to correspond to cells which, on visual inspection, showed a similar pattern of phase precession to the other cells, but for which the most significant regression line had a positive slope. Thus, many of these cells should probably be added to the proportions of ‘phase precessing’ cells in table 1.

**Figure 6.**
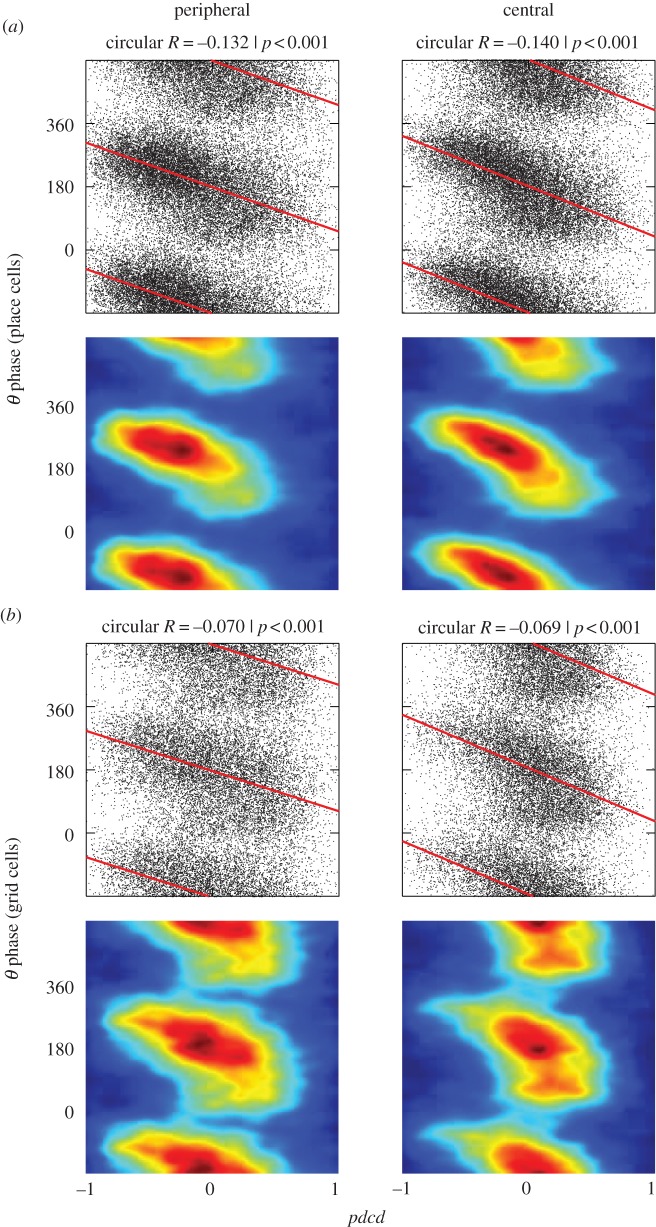
Phase precession versus *pdcd* (shown as in figure 5) in runs through the centre or periphery of the firing field. (*a*) Place cells. (*b*) Grid cells. (Online version in colour.)

In place cells (figure 4*a*), the rate of phase precession seems to increase on running through the centre of the firing field, compared with running through the edge of the firing field, but this pattern is less pronounced in grid cells (figure 4*c*). To investigate further, we examined spikes fired on runs through the centre of the firing field and runs through the periphery (figure 6). In both cases, robust phase precession is seen in both peripheral and central runs, although the slope of the relationship of phase to *pdcd* appears to be shallower in peripheral runs. The mean difference in the rate of phase precession between runs through the field centre compared with runs through the periphery is 66° per unit *pdcd* for grid cells (*p* < 0.001) and 82° for place cells (*p* < 0.0001; permutation test); see also [30].

## 4. Discussion

By normalizing the firing fields of place cells and grid cells of rats foraging in open environments, we were able to characterize the dependence of firing phase on some of the behavioural and physiological variables experienced during runs through the cell's firing field. We found that the best correlate of firing phase, of those variables tested, was the distance to the field peak projected onto the animal's current running direction (*pdcd*). Strong correlations were also found with other related measures of distance travelled through the firing field (*d*, *pdmd*). This pattern was true for both place cells and grid cells, highlighting the similarity of phase precession in both types of spatial cell. However, the overall strength of phase precession was greater for place cells than for grid cells.

The strong correlation of firing phase with the projected distance onto the current direction (*pdcd*) is consistent with previous observations of place cell firing phase in open environments [16] and with the correlation between place cell firing phase and ‘directional rate zones’ [17]. The directional rate zone measure involves a binary measure of whether the field peak is ahead of or behind the rat and uses the firing rate zone within the firing field as a proxy for distance from the peak. This is equivalent to our *pdcd* measure, given that our firing fields have been mapped onto a unit circle around the peak firing location, except that the cosine function used in our projection of distance onto current direction is replaced by a ±1 step function.

The correlation between phase and the time spent in the field during the run was weaker than with *pdcd*, owing in part to the variation in the rate of phase precession versus time across runs with slow and fast running speeds. Similarly, the correlation between firing phase and the number of spikes fired during the run (*spks*) was weaker than with *pdcd*, owing in part to the large variation in the slope of this relationship across high and low firing runs. It is possible that additional variance in the number of spike fired per run [42] weakened the relationship between phase and *spks*. However, we note that, although the correlations were weak, a larger overall proportion of place cells had a significant correlation between firing phase and *spks* than with any other variable (table 1). It may be that the relationship between phase and *spks* contributes to the relatively stable correlation of phase with distance travelled across fast and slow runs, as faster runs have higher firing rates. The mean in-field firing rate on the runs analysed is 4.4 Hz for phase precessing place cells, with a mean difference of 3.4 Hz between fast and slow runs (see also [43]). The mean in-field rate on the runs analysed is 4.8 Hz for grid cells, with a mean difference of 1.4 Hz between fast and slow runs; see also [36].

The stronger correlation between firing phase and distance rather than time supports the suggestion that the intrinsic firing frequency of place cells [15,22,43] and grid cells [23,44] increases with running speed to exceed the LFP theta frequency by more on fast runs than on slow runs. The relative inability of the number of spikes (*spks*) or expected amount of firing (*cfr*) on the run so far to explain changes in firing phase argues against models in which firing phase advances as a consequence of spiking dynamics, such as the time-course of after-hyperpolarization or after-depolarization (e.g. [21] for grid cells). However, models in which firing phase has a more complex relationship to previous spiking activity may provide a better fit to the data, and we note that a large proportion of place cells showed significant phase precession relative to the number of spikes fired (*spks*, table 1). The relatively weak correlation between phase and instantaneous firing rate (*ifr*) replicates a similar finding in place cells on linear tracks [40] and argues against simple mechanisms in which increased synaptic input directly relates to earlier firing phases [18–20] insofar as increased input is reflected in increased firing rates. Of course, more complex relationships between phase, firing rate and synaptic input may also provide a better fit to the data.

An interesting aspect of theta phase precession is that the correlation between firing phase and distance through the field is even more strongly present on each run through the field than in the data pooled across runs, for place cells [27] and especially for grid cells [28]. However, as noted in §1, it is the commonality across runs that is important in determining what can be inferred by a simple linear decoder. Equally, the rate of change of grid cell firing phase with distance is steeper within run than between runs because of spatial jitter in the overall set of firing locations from run to run [28]. This jitter needs to be subtracted from decoded locations run-by-run to be able to take advantage of the stronger within-run relationship between phase and distance. It will be interesting to see whether this spatial jitter is common across simultaneously recorded cells, which would aid its estimation and subtraction. Common spatial jitter in the location represented by grid cell firing is indicated in the analysis performed by [45].

The analysis of phase versus behavioural and physiological variables, pooled across runs, reflects the likely performance of a simple decoder more accurately than a run-by-run analysis would [27,28]. The different slopes of the relationship between firing phase and time, distance and spikes on different types of runs illustrates one aspect of this problem. A simple decoder of these variables from firing phase, i.e. one that does not know how to adjust the slope of the mapping from phase to *t*, *d* or *spks* run-by-run, will be unable to make good use of what might be strong correlations on any given run. For example, the relationship of firing phase with distance travelled and with time spent within the field might be equally strong within each run, but the pooled data indicate that the slope of the relationship with distance should be more constant across runs of different speed than the slope of the relationship with time.

We also characterized the two-dimensional spatial distribution of firing phase within the place field. Firing phase decreases from the first to second half of the firing field, and the variance of firing phase increases in the second half of the field (figure 4), consistent with previous analyses of place cells in open environments [10] and place [5,46] and grid cells [6] on linear tracks. The phase variance of grid cells seemed to be particularly high at the point at which the animal passed the peak of the firing field, consistent with the rapid change in projected distance (*pdcd*) at this point. There also appeared to be robust phase precession during runs through the centre and through the periphery of the field in both place and grid cells (figures 4 and 6). This is suggestive of the oscillatory interference model of grid cell firing [23–25,47,48], in which phase precession arises from ‘velocity-controlled oscillators’ (VCOs), each of which codes for the distance travelled along a given preferred direction. These VCOs provide the inputs to grid cells, and a given VCO drives grid cell phase precession when the rat is running along its preferred direction [24]. A consequence of this is that firing phase encodes the animal's location relative to the firing field projected onto the current running direction (as in *pdcd*), and occurs on peripheral or central runs through the grid field. The data show some signs of this effect: significant phase precession relative to *pdcd* in both peripheral and central runs, but also a tendency for shallower phase precession in peripheral runs. See [30] for further discussion of the comparison between oscillatory interference models and phase precession data.

In summary, the theta phase of firing of place cells and grid cells appears to represent the location of the animal within the currently occupied firing field. The way in which this location is represented during foraging in open environments is best captured by the distance of the field peak ahead of or behind the animal along its current direction of motion. As noted previously [16,49], this representation is directly useful for navigation. If a goal location is remembered by storing the pattern of place cell activity at the goal, then a simple model of navigation is to assume that the animal returns to the goal by moving to increase the match between the current pattern of place cell activity with the stored pattern. Within this simple model, the firing phase allows the animal to know whether its current direction is heading towards or away from a goal location by sampling locations ahead of or behind the animal within each theta cycle [49]; see also [50,51]. Within more complicated models, in which the goal location is sampled in different heading directions, the theta phase code would allow the storage of patterns of place cell firing representing locations displaced from the goal in different directions. This allows a population vector output during subsequent navigation which indicates the direction to the goal [16].
